# Robust *de novo* pathway enrichment with *KeyPathwayMiner 5*

**DOI:** 10.12688/f1000research.9054.1

**Published:** 2016-06-28

**Authors:** Nicolas Alcaraz, Markus List, Martin Dissing-Hansen, Marc Rehmsmeier, Qihua Tan, Jan Mollenhauer, Henrik J. Ditzel, Jan Baumbach

**Affiliations:** 1 Department of Mathematics and Computer Science, University of Southern Denmark, 5230 Odense, Denmark; 2 Department of Cancer and Inflammation Research, Institute of Molecular Medicine, University of Southern Denmark, 5000 Odense, Denmark; 3 Lundbeckfonden Center of Excellence in Nanomedicine NanoCAN, University of Southern Denmark, 5000 Odense, Denmark; 4 Institute of Clinical Research, University of Southern Denmark, 5000 Odense, Denmark; 5 Max Planck Institute for Informatics, 66123 Saarbrucken, Germany; 6 Integrated Research Institute (IRI) for the Life Sciences and Department of Biology, Humboldt-Universitat zu Berlin, 10099 Berlin, Germany; 7 Epidemiology, Biostatistics and Biodemography, Institute of Public Health, University of Southern Denmark, 5000 Odense, Denmark; 8 Department of Oncology, Odense University Hospital, 5000 Odense, Denmark

**Keywords:** Pathway enrichment, network analysis, data integration, algorithms, systems biology

## Abstract

Identifying functional modules or novel active pathways, recently termed de novo pathway enrichment, is a computational systems biology challenge that has gained much attention during the last decade. Given a large biological interaction network, KeyPathwayMiner extracts connected subnetworks that are enriched for differentially active entities from a series of molecular profiles encoded as binary indicator matrices. Since interaction networks constantly evolve, an important question is how robust the extracted results are when the network is modified. We enable users to study this effect through several network perturbation techniques and over a range of perturbation degrees. In addition, users may now provide a gold-standard set to determine how enriched extracted pathways are with relevant genes compared to randomized versions of the original network.

## Introduction

*De novo* pathway enrichment methods have gained much attention during the last decade due to their potential to identify novel regulators and putative biomarkers from vast datasets in systems biology research. Given a biological interaction network, such as defined by BioGrid ^1^, IntAct ^2^ or I2D ^3^, the main objective of *de novo* pathway enrichment is to extract connected subnetworks that are enriched for genes that are implicated in the phenotype of interest. This phenotype is dependent on the experiment and observed in one or several omics datasets, including, for instance, gene expression values, DNA methylation signals or single nucleotide variants. The common denominator of *de novo* pathway enrichment methods is that the resulting subnetworks are expected to include known pathways as well as novel pathways that have little overlap with annotated pathways found in curated databases. Existing approaches can be divided into the following categories: (i) aggregate score optimization methods, where the objective is to extract subnetworks that maximize a summary or statistical score of the individual gene scores, (ii) score propagation methods, where individual gene scores from the molecular profiles are propagated through the network, or adjusted to reflect also their connectivity in the network, (iii) module cover approaches, where the objective is to extract subnetworks containing genes that cover as many active cases/samples as possible, and (iv) cluster-based approaches. Methods that fall into categories (i), (ii) or (iv) rely heavily on the scoring function, which must be appropriate for the technology of the molecular profile being studied. In contrast, methods based on the module cover approach (iii) do not suffer from this issue, but leave it up to the user to find a sensible way to discern active from inactive genes. An overview of popular *de novo* pathway enrichment methods is shown in Table 1. We identify three issues common to existing *de novo* methods:

- There is little consensus on what constitutes a novel pathway. It is up to the user to find method-specific parameters that lead to a satisfying solution. Choosing these parameters is often not intuitive and even small changes can lead to large variations in the results. Most methods provide little guidance on parameter selection, forcing users to rely on educated guesses, or to tediously re-run the method multiple times until the optimal parameters for a given analysis are found.
- It has been demonstrated that for several methods results change significantly upon perturbations in the underlying networks ^4^. This lack of robustness is an issue, since interaction databases are continuously evolving and it is unclear to what degree the results will change when a particular tool is applied with the exact same data to a newer version of the network.
- In the rare cases where a ground truth or gold standard is available, the validation of *de novo* pathway enrichment results is not straightforward and, to our knowledge, not supported by any available method.

**Table 1.** A non-exhaustive selection of popular *de novo* network enrichment tools. Abbreviations: Cytoscape app (CA), standalone version/package (SA), desktop application (DA), web application (WA), web service (WS), visualization (VIZ), multi-omics (MO), robustness of the results upon network perturbation (RB), validation of the results using a gold standard upon network perturbation (VL).

	CA	SA	DA	WA	WS	VIZ	MO	RB	VL
BioNET ^5^	✘	✔	✘	✘	✘	✔	✘	✘	✘
GiGa ^6^	✘	✔	✘	✘	✘	✘	✘	✘	✘
GXNA ^7^	✘	✔	✘	✘	✘	✘	✘	✘	✘
HotNet ^8^	✘	✔	✘	✘	✘	✔	✘	✘	✘
jActiveModules ^9^	✔	✘	✘	✘	✘	✔	✘	✘	✘
MATISSE ^10^	✘	✘	✔	✘	✘	✔	✘	✘	✘
PinnacleZ ^11^	✔	✘	✘	✘	✘	✔	✘	✘	✘
RegMOD ^12^	✘	✔	✘	✘	✘	✘	✘	✘	✘
**KPM 5.0**	✔	✔	✘	✔	✔	✔	✔	✔	✔

We have previously developed KeyPathwayMiner, a *de novo* pathway enrichment tool following the module cover approach. Even though the parameters in KeyPathwayMiner are relatively intuitive, their selection becomes challenging for analyses involving several distinct omics datasets. To address this issue, we allow users to define a range (consisting of minimum, maximum and step size) for each parameter. The resulting grid search is fully automated and saves the user from going through tedious repetitions. While testing different parameters is more convenient in this way, it is still necessary to manually inspect the resulting subnetworks to select the optimal settings in a subjective fashion.

Here, we present version 5 of KeyPathwayMiner, which is the first tool to provide a user-friendly way to systematically evaluate the quality and robustness of the results in *de novo* pathway enrichment. We achieve this by perturbing the input network to varying degrees. Depending on the research question, several perturbation techniques are available. To assess robustness of the results, the largest solution found in the perturbed network(s) is compared against the largest solution found in the unperturbed network. The size and variance of the overlap is illustrated for different user-controlled levels of perturbation and is an indicator for the robustness of the results. If a gold standard is available, an additional measure of quality is the overlap of the largest solution found in the unperturbed as well as in the perturbed network(s) with the gold standard. As an example application case, we apply KeyPathwayMiner to a gene expression dataset covering 38 Huntington’s disease patients and 32 healthy controls. We demonstrate the potential of network perturbation to help assessing the quality and robustness of the extracted results.

## Methods

### Implementation

KeyPathwayMiner is implemented as a modular Java application centering on a core module that provides various *de novo* pathway enrichment strategies and methods for network perturbation analysis and plotting. A number of application modules have been implemented for different usage scenarios, including a standalone module, a web application module ^13^, and a Cytoscape app module. The web application module KeyPathwayMinerWeb ( http://keypathwayminer.compbio.sdu.dk), for instance, is primarily targeted at researchers with little to no experience in Cytoscape. No installation is necessary and convenience features, such as the mapping of identifiers or the conversion of p-value matrices to indicator matrices, are included. Web application developers may utilize a RESTful interface to integrate KeyPathwayMinerWeb seamlessly into their own applications. The standalone version is targeted at developers and data analysts who need more computational power than KeyPathwayMinerWeb offers and thus seek to incorporate KeyPathwayMiner directly in their own software implementation or in data analysis scripts, respectively. Finally, the Cytoscape app is the most powerful module, since it is also not limited with respect to the parameter range and computational power needed but also offers additional useful features such as combining OMICs datasets with a logical formula editor or the generation of plots using the JFreeChart ( www.jfreechart.org) library.

### Operation

**Figure 1.**
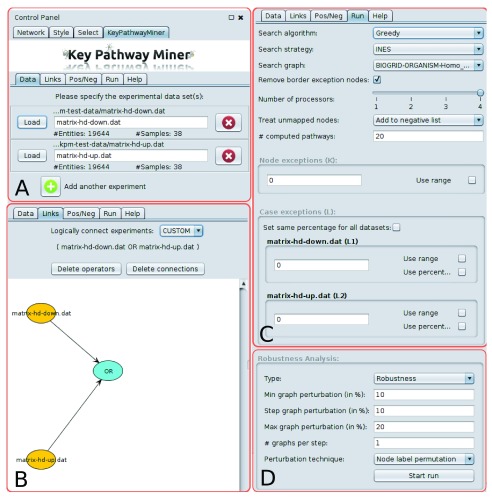
The user interface of the KeyPathwayMiner Cytoscape app is located in the control panel. The user sets the analysis up as follows (omitting the ‘Pos/Neg’ tab, where nodes can be specified for inclusion or exclusion): ( **A**) one or several dataset files are selected from the disk. ( **B**) Several dataset files can be logically connected via a formula editor. ( **C**) The run parameters are configured, most importantly the enrichment strategy (INES or GLONE), the search algorithm, the input network and the search parameters *K* and/or *L*, which can also be defined as a range. ( **D**) Network perturbation settings used in robustness or validation runs.

After installing the KeyPathwayMiner Cytoscape app via the app store, the user is expected to load an interaction network into Cytoscape. The KeyPathwayMiner tab can be found in the Control Panel and allows for one or more indicator matrices to be selected as input under the initial tab ‘Data’. These matrices can be derived from OMICs datasets such that samples correspond to columns and nodes (genes) to rows. Each entry in the matrix is either a ‘1’ indicating an active case in a node or ‘0’ otherwise. A typical example is a gene expression dataset in which a ‘1’ represents a differentially expressed gene. Another example could be a next-generation sequencing dataset where a ‘1’ indicates a single nucleotide polymorphism. Example files can be downloaded from the KeyPathwayMiner website at http://keypathwayminer.compbio.sdu.dk ( Figure 1A). In the next tab, called ‘Links’, the user can customize how several datasets are combined for the analysis. Here, one can choose between ‘AND’ (a case is considered active if it is active in all datasets), ‘OR’ (a case is considered active if it is active in any of the datasets), or ‘CUSTOM’, which allows for connecting datasets in an interactive formula editor ( Figure 1B). The tab ’Pos/Neg’ allows the user to define nodes that are always considered active (positive list) or that are ignored (negative list). In the ‘Run’ tab, it is finally possible to select the parameters for the KeyPathwayMiner run. Batch runs can also be performed by defining a range of values for *K* and *L*, such that users can conveniently run and assess the results for varying values of these parameters. ( Figure 1C). KeyPathwayMiner relies on two easy-to-interpret parameters to control the size of the extracted subnetworks. The user can choose between a local as well as a global enrichment strategy. In the local strategy, INEs (Individual Node ExceptionS), a gene is considered active when it is active in all but *L* cases/samples. In addition, a parameter *K* allows KeyPathwayMiner to add additional inactive genes to extend the size of the solutions. We observe that INES has a tendency to prefer hub nodes, which is not always desirable. We therefore implemented the GLONE (GLobal Node Exceptions) strategy, where the parameter *L* is considered across all genes and fewer hub nodes are selected at the cost of run-time.

The optimal values for *K* and *L* depend on the dataset ^14^. KeyPathwayMiner allows users to define a range for both parameters to identify the best settings in a straight-forward fashion.

Users can choose between different methods to extract subnetworks: an exact (fixed parameter tractable), a greedy, and a heuristic (ant colony optimization) algorithm. For additional details regarding KeyPathwayMiner we refer to ^14– 16^.

Several new features have been implemented in version 5 of KeyPathwayMiner and are described in the following.

### Network perturbation

KeyPathwayMiner now enables users to study the robustness and validity of the extracted subnetworks through perturbation ( Figure 1D). To this end, the user can choose from the following common strategies:

- Node label permutation: Pairs of nodes are selected arbitrarily and their node labels are swapped. This technique preserves the network structure exactly, but affects the local density of active genes in the network.
- Degree preserving rewiring: In this strategy first suggested by Maslov *et al.* ^17^, two arbitrary edges are selected and their endpoints are swapped. As a result, the local network structure is actively changed while the global topological structure and the node degree distribution remain intact. With a large number of permutations this strategy leads to a randomized network.
- Node removal: In this strategy, a certain percentage of arbitrarily selected nodes are removed, thus simulating what results on a less complete network would look like. This is particularly interesting since interaction networks are continuously growing in size.
- Edge removal: In contrast to node removal, which affects network size, this strategy affects primarily the density of a network.

### Robustness analysis

To assess the quality of the results, we consider the overlap of the largest solution between the various perturbed and the non-perturbed analyses. With an increasing degree of perturbation of the network, it can be expected that this overlap will decrease. Users can thus assess how robust the observed result is by considering the Jaccard similarity coefficient between the gene sets *S _perturbed_* and *S _unperturbed_* based on the gene sets extracted from the largest solution found using the perturbed and non-perturbed networks, respectively:

$$J(S_{perturbed},\;S_{unperturbed})=\frac{\left|S_{perturbed}\cap S_{unperturbed}\right|}{\left|S_{perturbed}\cup S_{unperturbed}\right|}\;(1)$$

### Validation analysis

Similarly, the comparison of the overlap of the largest solution of the perturbed as well as the non-perturbed analyses and a gold standard *S _Gold_* can be used as a quality metric:

$$J(S_{perturbed},\;S_{gold})=\frac{\left|S_{perturbed}\cap S_{gold}\right|}{\left|S_{perturbed}\cup S_{gold}\right|}\;(2)$$

$$J(S_{unperturbed},\;S_{gold})=\frac{\left|S_{unperturbed}\cap S_{gold}\right|}{\left|S_{unperturbed}\cup S_{gold}\right|}\;(3)$$

### New *L* parameter specification options

As a convenience feature, we now allow users to select *L*, which allows users to define the number of case exceptions allowed in a solution, to be defined as a percentage of the total number of cases. This is particularly advantageous in the case of multiple datasets, where the *L* parameter (range) can now be selected once for all datasets in spite of differences in the number of cases between them.

### Border exception node removal

**Figure 2.**
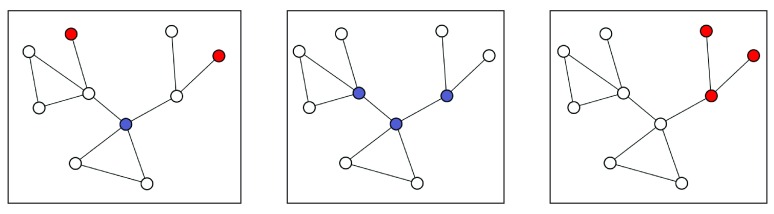
Three putative examples of solutions obtained with *K* = 3 exception nodes to illustrate the impact of border exception nodes (BENs). Removing BENs (red) would not disconnect regions containing no exception nodes (white). In contrast, removing non-BEN exception nodes (dark grey) would create two disjoint subnetworks containing non-exception nodes.

The INES model extracts subnetworks with up to *K* exception nodes that are not active or differentially expressed (as defined by the *L* parameter). In many cases, these exception nodes are central in the pathway, i.e. they connect (groups of) active genes. However, if the *K* parameter is too large, some of these exception nodes are simply added to the periphery of the subnetwork to increase the size of the solution ( Figure 2). As a result, the top solutions of a KeyPathwayMiner run would sometimes consist of overlapping subnetworks that only differ in these border exception nodes (BENs). BENs can now be removed in an optional filtering step, which will lead to more diverse solutions.

BENs are removed as shown in Algorithm 1, which has worst-case running time of *O*((| *V*| + | *E*|) ∗ *K*).

## Use cases

We tested the usability of the new KeyPathwayMiner features with a gene expression dataset consisting of tissue samples from the caudate nucleus region of the brain ^18^ taken from 38 patients suffering from Huntington’s disease (HD) and from 32 healthy patients in the control group. While it is known that huntingtin protein plays a major role in the development of the disease, the corresponding gene is not differentially expressed in approximately 40% of the patients. Hence, it will not be found in an analysis focused on identifying differentially expressed genes. However, it can be expected that protein-protein interaction (PPI) partners of the huntingtin protein are differentially expressed on the transcript level, thus posing an ideal test case for KeyPathwayMiner, which can identify subnetworks with huntingtin as an exception node. The Human Protein Reference Database (HPRD version 9, http://www.hprd.org/download) ^19^ was used as the interaction network. To produce an indicator matrix for down-regulation, a one-tailed t-test was used to compute p-values for each gene and patient in the disease group vs all patients in the control group. Afterwards, a p-value cutoff of 0.05 was selected to set a 1 (significant) or 0 (not significant) in the indicator matrix for down-regulation (file available at http://keypathwayminer.compbio.sdu.dk/downloads/matrix-hd-down.dat).

In a first use case we performed a robustness analysis with KeyPathwayMiner (INES model, greedy algorithm) by fixing parameters to *L* = 20% (8 out of 32) of the cases and *K* = 5 exception nodes. In other words, we searched for maximal connected subgraphs containing at most 5 nodes with no more than 20% of cases in which the gene represented by the network node is not down-regulated.

**Algorithm 1. Border exception node filter**

    
**Input**: Graph *G*( *V*, *E*), Exception Nodes *V _e_* ⊂ *V*

    
**Output**: Subgraph *G*′ ⊆ *G* without BENs

    
*G*′ := *G* ;

    
**while** Ve≠0 **do**

         
Vben:=0 ;

         
*v* := select and remove random node from *V _e_* ;

         
*E _v_* := edges incident to *v* ;

         
*G _temp_* := *G*′( *V* \ { *v*}, *E* \ *E _v_*) ;

         
*C* := connected components of *G _temp_* ;

         
*s* := 0 ;

         
**foreach** *c* ∈ *C* **do**

              
**if** *V*( *c*) ∩ *V _e_* == *V*( *c*) **then**

                   
*V _ben_* := *V _ben_* ∪ *V*( *c*)

              
**else**

                   
*s* := *s* + 1 ;

              
**end**

         
**end**

         
**if** *s* == 1 **then**

              
*V _ben_* := *V _ben_* ∪ { *v*}

         
**end**

         
*V _e_* := *V _e_* \ *V _ben_* ;

         
*E _ben_* := edges incident to *V _ben_* in *G*′ ;

         
*G*′ := *G*′( *V* \ *V _ben_*, *E* \ *E _ben_*) ;

    
**end**

    
**return** *G*′ ;

In a typical robustness scenario, we wanted to test how the solutions change when a certain percentage of the edges in the graph are removed randomly. We thus selected "edge removal" as the perturbation technique with perturbation levels selected to range from 10% to 50% in increments of 10%. For each perturbation level, we created 10 randomly perturbed networks and executed KeyPathwayMiner with identical settings as in the original run.

**Figure 3.**
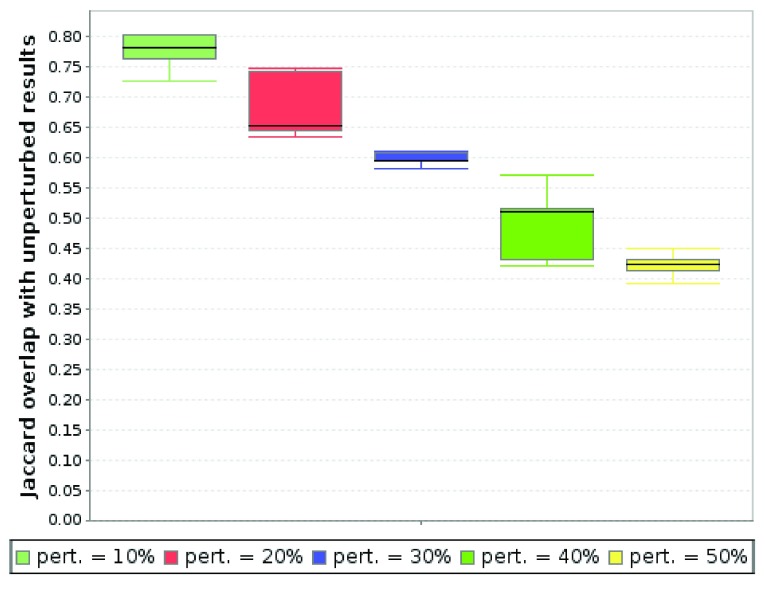
Robustness results for different percentages of edge removal. For each perturbation level, 10 different networks were generated randomly and submitted to KeyPathwayMiner for analysis. (Parameters: INES, greedy, *K* = 5, *L* = 20%)

As one would expect, removing a certain percentage of edges reduced the overlap with the results from the original network. However, even after removing 50% of the edges ( Figure 3), a moderate Jaccard index overlap of 0.45 was observed.

In a second use case, we give an example of a validation run. To this end, we compiled a gold standard gene set consisting of HD relevant genes (file available at http://keypathwayminer.compbio.sdu.dk/downloads/htt-relevant.txt) from the KEGG ^20, 21^ HD and calcium signaling pathways. Calcium signaling has been suggested to have an important role in the development of HD ^22^.

**Figure 4.**
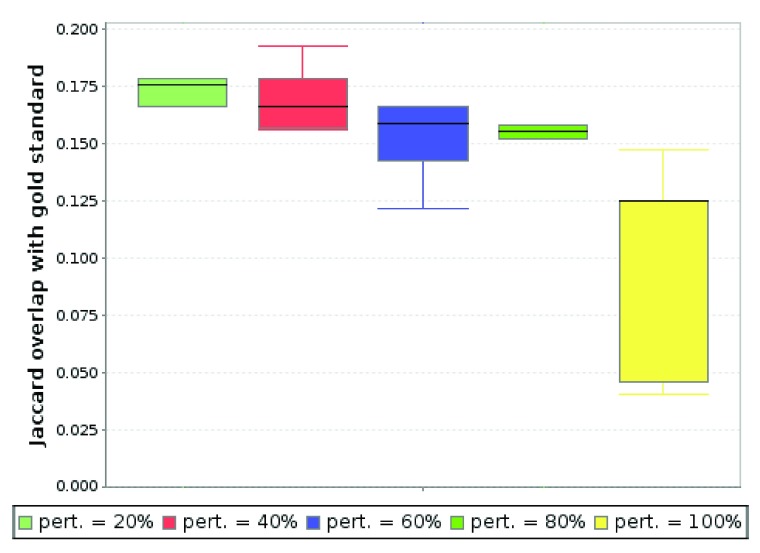
Overlap with the selected HD gold standard gene set with different percentages of permuted node labels in the input network. For each perturbation degree, 10 graphs were generated. (Parameters: INES, greedy, *K* = 5, *L* = 15%). Note that partial network perturbation and subsequent comparison with gold standard sets has limited meaning. Biological interaction networks are scale-free, i.e. robust to random perturbations. Of major importance for this effect are a small number of hub nodes. KeyPathwayMiner is able to recover subnetworks containing relevant genes connected to such hubs unless the hubs themselves are affected by the perturbation. This, however, is only the case when 100% of the network is perturbed (a randomized, true null model), explaining the performance drop we observe for this degree of perturbation.

In this scenario we wanted to see how solutions overlap with gold standard gene sets when randomly shuffling the node labels. In addition to the indicator matrix for down-regulation, we also aimed at finding solutions containing up-regulated genes. Hence we produced an additional indicator matrix for up-regulation (file available at http://keypathwayminer.compbio.sdu.dk/downloads/matrix-hd-up.dat) and connected them both with an ‘OR’ operator. We set a common *L* = 15% for both sets together with *K* = 5. KeyPathwayMiner (INES model, greedy algorithm) thus searched for pathways containing at most five genes with at most *L* = 15% genes that are not differentially regulated. The perturbation technique chosen was "node label permutation". The results show that even when permuting 80% of the node labels the overlap with the gold standard set remains relatively stable. As expected, we can see a significant drop when all labels are permuted ( Figure 4).

Use case data of *de novo* pathway enrichment with KeyPathwayMiner 5 Data from use cases are provided. Please see text file for a description of each set of data. Click here for additional data file. Copyright: © 2016 Alcaraz N et al. 2016 Data associated with the article are available under the terms of the Creative Commons Zero "No rights reserved" data waiver (CC0 1.0 Public domain dedication).

## Summary

*De novo* pathway enrichment is a powerful method for the analysis of one or several types of molecular profiles. In contrast to widely used gene set enrichment methods such as GSEA ^23^, this methodology is not limited to existing knowledge but suitable to uncover new functional modules. Results are extracted using large biological interaction networks, which are incomplete and continuously evolve. It is typically unclear how future updates leading to an interaction network of higher quality will affect the currently obtained results. KeyPathwayMiner 5 enables users to study the robustness of their results by allowing them to introduce artificial noise into the underlying interaction networks. Moreover, an existing gold standard can be used to test how well the optimal solution can be recovered on these perturbed networks.

## Data availability

The data referenced by this article are under copyright with the following copyright statement: Copyright: © 2016 Alcaraz N et al.

Data associated with the article are available under the terms of the Creative Commons Zero "No rights reserved" data waiver (CC0 1.0 Public domain dedication). 

F1000Research: Dataset 1. Use case data of *de novo* pathway enrichment with KeyPathwayMiner 5, 10.5256/f1000research.9054.d126871 ^24^

## Software availability

1. Software available from: http://apps.cytoscape.org/apps/keypathwayminer
2. Latest source code: https://github.com/baumbachlab/keypathwayminer-cytoscape3 https://github.com/baumbachlab/keypathwayminer-cytoscape3/archive/5.0.tar.gz (KeyPathwayMiner Cytoscape app source code) https://github.com/baumbachlab/keypathwayminer-core/archive/5.0.tar.gz (KeyPathwayMiner core library source code)
3. Archived source code as at time of publication: Zenodo, Source codes *de novo* pathway enrichment with KeyPathwayMiner, doi: 10.5281/zenodo55734 ^25^
4. License: GPL v3
