# Supplementary material for: Robust de novo pathway enrichment with KeyPathwayMiner 5
Source: F1000Res. 2016 Jun 28;5:1531. [Version 1] doi: 10.12688/f1000research.9054.1 (PMC4965696; doi:10.12688/f1000research.9054.1)
Supplement: Peer review history

# Peer review: Referee response for version 1

**DOI:** 10.5256/f1000research.9744.r15263

**Published:** 2016-07-28

**Authors:** Mona Riemenschneider

**Affiliations:** 1 Department of Bioinformatics, Straubing Center of Science, Weihenstephan-Triesdorf University of Applied Science, Straubing, Germany

The manuscript describes a tool for robust *de novo* pathway enrichment. The software provides the great advantage to study effects of network perturbations thereby allow for the evaluation of quality and robustness of the results in *de novo* pathway enrichment. Thus, the authors address a relevant issue in network construction and pathway enrichment.

 The rationale for the development of the tool is clearly stated. A use case to demonstrate the usability of KeyPathwayMiner 5 with varying parameters is described within the manuscript. The source code of KeyPathwayMiner 5 is available at github.com.

 MINOR COMMENTS:

 The introduced tool KeyPathwayMiner 5 is a further development of KeyPathwayMiner x. A short overview of added features to all updated versions may be helpful for users to get an overview of the full function of KeyPathwayMiner 5. (provide in supplement)

 Several parameters must be set to run KeyPathwayMiner. Could an approximate recommendation for parameter values and settings be given for non-expert users?

 The calculation of the Jaccard index is given in detail, however a short explanation of the graduation (high, moderate, low) of calculated values could be helpful to provide easy interpretation of results for all users.

 Please check spelling throughout the manuscript: KeyPathwayMiner/KeyPathway Miner

I have read this submission. I believe that I have an appropriate level of expertise to confirm that it is of an acceptable scientific standard.

# Peer review: Referee response for version 1

**DOI:** 10.5256/f1000research.9744.r14664

**Published:** 2016-07-01

**Authors:** Alberto Calderone

**Affiliations:** 1 Bioinformatics and Computational Biology Unit, Molecular Genetics Laboratory - Tor Vergata University, Rome, Italy

The article presents an update to a previously published Cytoscape App by providing a general overview of other methods. The title, as well as the abstract give a good introduction to the article.

 The authors give a good recap of other methods and possible approaches as well as presenting the updated app. The Cytoscape app presentation is clear and enough for the final user. Most importantly, the new feature introduced in V5 - i.e. Perturbation - is clearly described in a dedicated paragraph. "Robust" in the title is justified in the text and use cases were clear and doable. Border exception node is illustrated clearly as well.

 I could reproduce the examples given with no big problem. Overall, the article is well written.

I have read this submission. I believe that I have an appropriate level of expertise to confirm that it is of an acceptable scientific standard.
